# Identification of a Core Set of Genes That Signifies Pathways Underlying Cardiac Hypertrophy

**DOI:** 10.1002/cfg.428

**Published:** 2004

**Authors:** Claes C. Strøm, Mogens Kruhøffer, Steen Knudsen, Frank Stensgaard-Hansen, Thomas E. N. Jonassen, Torben F. Ørntoft, Stig Haunsø, Søren P. Sheikh

**Affiliations:** 1 CHARC (Copenhagen Heart Arrhythmia Research Center) Department of Medicine B, H : S Rigshospitalet University of Copenhagen Medical School 20 Juliane Mariesvej Copenhagen DK 2100 Denmark; 2 Molecular Diagnostic Laboratory Department of Clinical Biochemistry Aarhus University Hospital Denmark; 3 Center for Biological Sequence Analysis Technical University of Denmark Lyngby Kgs 2800 Denmark; 4 Department of Pharmacology Copenhagen University Copenhagen 2100 Denmark

## Abstract

Although the molecular signals underlying cardiac hypertrophy have been the subject of intense investigation, the extent of common and distinct gene regulation between different forms of cardiac hypertrophy remains unclear. We hypothesized that a general and comparative analysis of hypertrophic gene expression, using microarray technology in multiple models of cardiac hypertrophy, including aortic banding, myocardial infarction, an arteriovenous shunt and pharmacologically induced hypertrophy, would uncover networks of conserved hypertrophy-specific genes and identify novel genes involved in hypertrophic signalling. From gene expression analyses (8740 probe sets, *n* = 46) of rat ventricular RNA, we identified a core set of 139 genes with consistent differential expression in all hypertrophy models as compared to their controls, including 78 genes not previously associated with hypertrophy and 61 genes whose altered expression had previously been reported. We identified a single common gene program underlying hypertrophic remodelling, regardless of how the hypertrophy was induced. These genes constitute the molecular basis for the existence of one main form of cardiac hypertrophy and may be useful for prediction of a common therapeutic approach. Supplementary material for this article can be found at: http://www.interscience.wiley.com/jpages/1531-6912/suppmat


					Comparative and Functional Genomics
Comp Funct Genom 2004; 5: 459-470.
Published online in Wiley InterScience (www.interscience.wiley.com). DOI: 10.1002/cfg.428
Research Article
Identification of a core set of genes that
signifies pathways underlying cardiac
hypertrophy
Claes C. Strom1, Mogens Kruhoffer2, Steen Knudsen3, Frank Stensgaard-Hansen1, Thomas E. N. Jonassen4,
Torben F. Orntoft2, Stig Haunso1 and Soren P. Sheikh1*
1CHARC (Copenhagen Heart Arrhythmia Research Center), Department of Medicine B, H : S Rigshospitalet, University of Copenhagen
Medical School, 2100 Copenhagen, Denmark
2Molecular Diagnostic Laboratory, Department of Clinical Biochemistry, Aarhus University Hospital, Denmark
3Center for Biological Sequence Analysis, Technical University of Denmark, 2800 Kgs, Lyngby, Denmark
4Department of Pharmacology, Copenhagen University, 2100 Copenhagen, Denmark
*Correspondence to:
Soren P. Sheikh, Lab. Mol. Card.,
Department of Medicine B, H : S
Rigshospitalet, University of
Copenhagen, 20 Juliane
Mariesvej, DK-2100
Copenhagen, Denmark.
E-mail: sheikh@molheart.dk
Received: 6 February 2004
Revised: 30 August 2004
Accepted: 21 September 2004
Abstract
Although the molecular signals underlying cardiac hypertrophy have been the
subject of intense investigation, the extent of common and distinct gene regulation
between different forms of cardiac hypertrophy remains unclear. We hypothesized
that a general and comparative analysis of hypertrophic gene expression, using
microarray technology in multiple models of cardiac hypertrophy, including aortic
banding, myocardial infarction, an arteriovenous shunt and pharmacologically
induced hypertrophy, would uncover networks of conserved hypertrophy-specific
genes and identify novel genes involved in hypertrophic signalling. From gene
expression analyses (8740 probe sets, n = 46) of rat ventricular RNA, we identified a
core set of 139 genes with consistent differential expression in all hypertrophy models
as compared to their controls, including 78 genes not previously associated with
hypertrophy and 61 genes whose altered expression had previously been reported.
We identified a single common gene program underlying hypertrophic remodelling,
regardless of how the hypertrophy was induced. These genes constitute the molecular
basis for the existence of one main form of cardiac hypertrophy and may be useful
for prediction of a common therapeutic approach. Supplementary material for this
article can be found at: http://www.interscience.wiley.com/jpages/1531-6912/suppmat
Copyright  2004 John Wiley & Sons, Ltd.
Keywords: cardiac hypertrophy; remodelling; gene expression; DNA microarrays
Introduction
Cardiac hypertrophy is a compensatory mecha-
nism to augment cardiac output after biomechani-
cal stress. Sustained hypertrophy leads to cardiac
dysfunction, heart failure and arrhythmia, hence
hypertrophy is an independent risk factor for car-
diac morbidity and mortality (Agabiti-Rosei et al.,
1997). Understanding the genes that govern car-
diomyocyte hypertrophic properties has implica-
tions for both clinical medicine and basic cell biol-
ogy.
Cardiac hypertrophy appears in different phe-
notypes, depending on the eliciting stimuli; how-
ever, it is unclear whether this is the result of
divergent transcriptional responses or if a common
hypertrophy gene program exists (Aronow et al.,
2001; Chien et al., 1991; Wollert et al., 1996). The
structural changes in the left ventricle including
eccentric and concentric hypertrophy can be mim-
icked in animal models by volume and pressure
overload procedures, respectively. Eccentric hyper-
trophy is characterized by left ventricular dilation
Copyright  2004 John Wiley & Sons, Ltd.
460 C. C. Strom et al.
and decreased wall thickness, while in concentric
hypertrophy the ventricular wall is thickened and
the left ventricle reduced. Typically, arteriovenous
shunts and myocardial infarction induce eccentric
hypertrophy, while aortic banding elicits the con-
centric phenotype.
Multiple genes change expression in cardiac
hypertrophy including genes involved in metabo-
lism, cell growth, apoptosis, cytoskeletal and extra-
cellular matrix (ecm) organization, signal transduc-
tion and cell defence (Anversa et al., 1983; Barrans
et al., 2002; Friddle et al., 2000; Hwang et al.,
2000, 2002; Schoenfeld et al., 1998; Sehl et al.,
2000; Stanton et al., 2000; Yang et al., 2000).
Several genes are bona fide molecular markers
for hypertrophy, including immediate early genes,
ANP, BNP, and myosin heavy and light chain iso-
forms.
However, a comprehensive analysis and direct
comparison of the expression changes in different
forms of cardiac hypertrophy is lacking. Such an
approach may identify shared molecular networks
in hypertrophy and point to new therapeutic strate-
gies that interrupt the underlying disease pathways.
DNA microarrays allow simultaneous analysis of
thousands of genes, and have been useful in analy-
sis of cellular responses to different stimuli, animal
models of human disease and cancer classification
(Golub et al., 1999; Roberts et al., 2000). Using
this technology, we attempted to define a unified
gene response that characterizes cardiac hyper-
trophy by comparing transcriptional profiles from
multiple models, including aortic banding, myocar-
dial infarction, arteriovenous shunting and pharma-
cologically induced hypertrophy. Our results pro-
vide a detailed view of the shared gene expression
patterns in different hypertrophic phenotypes. Out
of 8740 analysed genes on 46 microarrays, we iden-
tified a set of 139 genes with common regulation
in all hypertrophy models, supporting the notion of
a common hypertrophic gene program.
Methods
Cardiac hypertrophy models
Male Wistar rats were used in all experiments. All
operations were performed during anaesthesia with
fentanyl/fluanisone and midazolam. The investiga-
tion conforms to the Guide for the Care and Use of
Laboratory Animals, published by the US National
Institutes of Health (NIH Publication No. 85-23,
revised 1996). Animals were subjected to echocar-
diographic examination under isoflurane anaesthe-
sia before being sacrificed. Thereafter, the hearts
were excised, rinsed in ice-cold saline, weighed,
dissected into left and right ventricles, frozen in
liquid nitrogen and stored at -80 
C before mRNA
extraction.
Neonatal hearts were harvested 3 days postpar-
tum.
Aortic banding (AB)
A titanium clip with an inner diameter of 0.6 mm
was placed around the ascending aorta, using a
custom-made applicator (Weck Closure Systems,
USA). Control animals underwent the same proce-
dure except for placement of the clip. The animals
were sacrificed after 6, 12, 16 or 30 weeks.
Myocardial infarction (MI)
The LAD coronary artery was occluded with a 6-
0 silk ligature immediately below the left atrial
appendage. In sham-operated animals, the ligature
was passed under the artery and removed. Animals
were sacrificed 3 or 9 weeks after surgery. Only
hearts with large transmural infarctions were con-
sidered for microarray analysis. The scar tissue was
removed and discarded.
Aorto-caval fistula (shunt)
The aorta was punctured caudal to the left renal
artery with an 18-gauge needle that was subse-
quently advanced into the vena cava. The needle
was withdrawn and the aortic puncture point was
sealed with cyanoacrylate glue. The patency of the
shunt was verified visually before closure. Control
animals underwent the same procedure except for
puncture of the vessels. The animals were sacrificed
after 3 or 8 weeks.
Hormone treatment
Angiotensin II (AngII; 200 ng/kg/min) was admin-
istered subcutaneously for 2 weeks, using implan-
ted miniosmotic pumps (ALZET
, USA). AngII
was diluted in 0.9% NaCl with 0.01 M acetic
acid. Age-matched control animals received pumps
containing vehicle only. The thyroxin analogue
Copyright  2004 John Wiley & Sons, Ltd. Comp Funct Genom 2004; 5: 459-470.
Gene expression in cardiac hypertrophy 461
3,5-diiodothyropropionic acid (Ditpa) was adminis-
tered as daily subcutaneous injections of 1000 mg/
kg. Controls were given saline.
Echocardiography
Echocardiography was performed during anaesthe-
sia with 1-1.5% isoflurane using a Vivid Five
Echocardiograph from GE Medical Systems Ultra-
sound. Recordings were stored digitally for off-line
analysis. Left ventricular cavity and wall dimen-
sions were measured in 2D short axis recordings.
Gene expression profiling
GeneChip RGU34A from Affymetrix containing
8740 genes (and 59 control genes, which were
excluded from further analysis) was used for all
hybridizations. Approximately 6000 known genes
are represented on the chip, the rest being ESTs (see
www.affymetrix.com for a more detailed descrip-
tion). Standard protocols for chip hybridizations
were used. At each time point in each model,
both diseased and control animals were randomly
split into two to four groups, and RNA isolated
from the left ventricle from animals in each group
were pooled and hybridized to the oligonucleotide
arrays. Thus, each time point in each model is
represented by four to six (two to four diseased,
and two controls) independent RNA pools, each
hybridized to an array, in total 46 arrays. Raw
data are available at www.ncbi.nlm.nih.gov/geo as
Series No. GSE 738.
Array data analysis
Array data were normalized using the non-linear
invariant rank fitting method of Li and Wong, avail-
able at www.dchip.org (Li et al., 2001a, 2001b).
Model-based expression values (MBE) were cal-
culated for each gene using dChip (perfect match
only model). For each hypertrophic sample, a log
fold change was calculated for each gene by divid-
ing the MBE value by the average MBE value of
the two relevant control samples and then applying
log2. This approach was taken to minimize variance
not related to hypertrophy between samples.
Statistical significance
Genes with common regulation in different hyper-
trophic samples were identified using Wilcoxon's
rank sum test on the log fold changes, while
ANOVA was used to identify differences between
the models. The p values calculated for each gene
were Bonferroni corrected by discarding all genes
with a p value higher than 0.05 divided by the
number of genes. The Bonferroni correction is a
method to control type 1 error when doing mul-
tiple comparisons. The principle is to divide the
desired type 1 error (usually 0.05) by the number
of comparisons.
Clustering
For calculation of distances between genes, fold
change was calculated by divided individual MBE
by the average MBE of all control samples and
applying log2. Vector angle distance on means nor-
malized data is identical to the Pearson correlation.
So the only difference between Pearson correlation
and vector angle is a normalization step. Hierar-
chical clustering was applied (weighted pair-group
average linkage) and visualized using ClustArray
(C. Workman, unpublished; www.cbs.dtu.dk).
Real-time PCR
cDNA was synthesized from single samples pre-
viously analysed on GeneChips. Reverse transcrip-
tion was performed using Superscript II RT (Invit-
rogen). 1 g total RNA and 1 l 50 pmol/l (dT)24-
primer in a total volume of 12 l was incubated for
10 min at 70 
C and chilled on ice. After adding
4 l 1st Strand Buffer (from supplier), 1 l DTT
(0.1 M), 2 l dNTP mix (10 mM) and 1 l Super-
Script RT II (200 U/l), the reaction was incubated
for 1 h at 42 
C and finally for 5 min at 95 
C.
The cDNA was diluted 1 : 20 for use in real-time
PCR. Real-time PCR analysis was performed on
selected genes using the primers shown in Figure 1.
Primers were designed using the Primer3 soft-
ware available at www-genome.wi.mit.edu/cgi-
bin/primer/primer3 www.cgi. Triple determina-
tions were performed on the ABI PRISM
7000
Sequence Detection System using the SYBR
Green PCR Master Mix (Applied Biosystems,
USA). To determine the relative gene expression, a
standard curve was made with serial dilutions of a
reference sample. The gene expression in the sam-
ple of interest was then determined relative to the
expression in the reference sample via the standard
curve (built-in feature of the analysis software, ABI
Copyright  2004 John Wiley & Sons, Ltd. Comp Funct Genom 2004; 5: 459-470.
462 C. C. Strom et al.
Chip PCR
Chip PCR
Chip PCR
Chip PCR Chip PCR
Chip PCR Chip PCR
Chip PCR
-1.0
0.0
1.0
2.0 o
o
o
o
o
o
o
o
o
o
o
o
o
o
o
o
o
o
ACTA1
Log
2
(fold
change)
-2.0
-1.0
0.0
o
o
o
o
o
o
o
o
o
o
o
o
o
o
o
o
o
o
AQP7
0.0
0.5
1.0
1.5
o
o
o
o
o
o
o
o
o
o
o
o
o
o
o
o
o
o
ARGBP2
0.0
1.0
2.0
3.0
o
o
o
o
o
o
o
o
o
o
o
o
o
o
o
o
o
o
CSRP2
0.0
0.5
1.0
1.5
o
o
o
o
o
o
o
o
o
o
o
o
o
o
o
o
o
o
FSTL1
Log
2
(fold
change)
o
o
o
o
o
o
o
o
o
o
o
o
o
o
o
o
o
o
NPPA
0.0
1.0
2.0
o
o
o
o
o
o
o
o
o
o
o
o
o
o
o
o
o
o
S100A4
0.5
1.0
1.5 o
o
o
o
o
o
o
o
o
o
o
o
o
o
o
o
o
o
TSC22
Log
2
(fold
change)
ACTA1: gctcttccagccttccttta
gcgtacaggtccttcctgat
CSRP2: ctcaacctcacaggcctaca
tgatcttctcagcagcatacac
ARGBP2: agaaccgtttcaggctctgt
gaaccatccgtcatcacact
FSTL1: atccttcaaccctcctgaga
aggaacagacacagcgattg
Probe pairs
NNPA: aggagaagatgccggtagaa
cacctcagagagggagctaag
S100A4: gctgaacaagacagagctcaa
cctgttgctgtccaagttgt
TSC22: ctgaaggagcagatcaaagaac
tggccagtgtcttcaacag
AQP7: gcatccttgtttgcgttct
gccagcaatgaaagtgaaga
2
3
4
5
Figure 1. Expression of selected genes was confirmed by real-time PCR. The fold changes of individual genes were
calculated as the log2 ratio of expression values between hypertrophic samples and their controls. Individual data points
are represented by open circles and no change in expression is indicated by grey lines. Means are shown by a cross and
SEM by arrows
Prism 7000 SDS Software 1.0.1). The PCR reaction
consisted of 12.5 l SYBR Green PCR Master Mix,
300 nM forward and reverse primers, and 2.5 l
1 : 20 diluted template cDNA in a total volume
of 25 l. The reaction was thermocycled using the
default settings of ABI Prism 7000 SDS Software
1.0.1: 2 min at 50 
C, 10 min at 95 
C, followed
by 40 rounds of 15 s at 95
C and 1 min at 60 
C.
A dissociation protocol was added after thermocy-
cling, determining dissociation of the PCR products
from 65 
C to 95 
C. All samples were normalized
to GAPDH. For each sample a triple determina-
tion was made for each gene of interest and the
normalization gene (GAPDH). An average was cal-
culated for the triple determinations. To normalize
the gene expression for a specific sample, the aver-
age expression value was divided by the average
expression value for the normalization gene in the
same sample. According to the GeneChip data,
GAPDH is consistently expressed in our samples.
Signals from GeneChip analyses were compared to
the normalized real-time PCR data.
Results
Animal models for cardiac hypertrophy
All experimental models of cardiac hypertrophy
resulted in significant left ventricular hypertrophy
(Table 1). In animals with a myocardial infarc-
tion (MI), echocardiographic examination revealed
massive dilation of the left ventricular cavity,
thinning of the anterior wall due to infarction,
and decreased systolic function (Table 2). Pressure
overload induced by aortic banding (AB) resulted
Copyright  2004 John Wiley & Sons, Ltd. Comp Funct Genom 2004; 5: 459-470.
Gene expression in cardiac hypertrophy 463
Table 1. Heart weights
Weeks n HWMI LVMI
Aortic banding 6 6 5.0  0.16 3.7  0.14
Sham 6 6 3.0  0.06 2.0  0.04
Aortic banding 12 6 4.0  0.26 2.9  0.17
Sham 12 6 2.5  0.04 1.7  0.01
Aortic banding 16 8 4.3  0.17 3.0  0.08
Sham 16 6 2.6  0.07 1.7  0.03
Aortic banding 30 8 4.3  0.37 2.9  0.20
Sham 30 6 2.3  0.09 1.5  0.06
MI 3 19 3.7  0.10 2.2  0.05
Sham 3 6 3.0  0.06 1.9  0.04
MI 9 14 3.5  0.12 2.1  0.04
Sham 9 6 2.6  0.07 1.7  0.04
Shunt 3 10 4.0  0.19 2.5  0.08
Sham 3 6 2.8  0.05 1.9  0.03
Shunt 8 12 4.0  0.23 2.5  0.11
Sham 8 6 2.7  0.07 1.8  0.04
Angiotensin II 2 12 3.1  0.04 2.1  0.05
Ditpa 2 11 3.2  0.1 2.2  0.10
Vehicle 2 21 2.8  0.04 1.9  0.03
Values are listed as mean  SEM. Heart weights (HW) and left
ventricular (LV) weights were divided by body weights (HWMI
and LVMI). Other abbreviations used: 3,5-diiodothyropropionic acid
(Ditpa), myocardial infarction (mi),  p < 0.05 vs. sham/vehicle.
in wall thickening but no dilation of the left ventric-
ular cavity consistent with concentric hypertrophy.
Systolic function was preserved in these animals.
Volume overload induced by a shunt between the
aorta and the caval vein was characterized by ven-
tricular dilation and no or marginal increases in
wall thickness consistent with eccentric hypertro-
phy. Systolic function was marginally depressed in
the volume-overloaded animals.
A unified gene expression response to cardiac
hypertrophy
To identify genes with a common gene expres-
sion pattern in hypertrophy, we analysed cardiac
gene expression by DNA microarrays. After nor-
malization to the appropriate controls, consistent
gene expression changes between the different phe-
notypes of cardiac hypertrophy were identified by
comparing gene expression changes in the different
models using a Wilcoxon test. To avoid false pos-
itives as a result of multiple testing, p values were
Bonferroni-corrected, such that the risk of one or
more false positive results was less than 5%.
This procedure identified 179 genes, representing
139 known genes, 13 ESTs and 27 gene duplicates
that were significantly regulated in hypertrophic
hearts vs. controls (Table 3 in this paper, and
Supplemental Table A). Among the 179 genes, 137
were upregulated and 42 downregulated. Of the 139
known genes, 61 have previously been reported
to change expression in response to hypertrophy,
while 78 have not previously been connected with
hypertrophy. A literature search revealed that 30
of the 137 genes have previously been reported
to change at the protein level (Supplemental Table
A). This confirms that many of the transcriptome
changes actually result in changed cellular protein,
considering that only 61 genes had previously been
associated with hypertrophy.
Figure 2 depicts a hierarchic cluster analysis
of the commonly regulated genes. All duplicate
genes were equally regulated and clustered tightly
together. The genes fell into two major clusters,
representing up- and downregulated genes, respec-
tively.
Classification into seven major categories
based on biological function revealed that the
core hypertrophy-related genes were predomi-
nantly involved in metabolism, signal trans-
duction, cytoskeletal/ecm organization, and cell
defence/inflammation (Figure 3). Most notably, in
the cluster of downregulated genes (Figure 2) the
vast majority of genes were involved in -oxidation
of fatty acids and oxidative phosphorylation.
Cardiac hypertrophy is associated with exten-
sive remodelling of the extracellular matrix. In line
with this notion, we observed enhanced expres-
sion of several ecm genes previously associated
with hypertrophy (fibronectin, vimentin, biglycan,
tissue-inhibitor of metalloproteinase-1 and -2, and
osteopontin) and genes not previously connected
with myocardial remodelling (protease nexin-1,
profilin-1 and osteoprotegerin). Several cytoskele-
tal genes were also upregulated (skeletal and vascu-
lar smooth muscle -actin). Several genes involved
in inflammation were upregulated (Hsp27, Psme1,
Pai1, and several complement genes), confirming
the notion that inflammation is important in the
hypertrophic process.
Genes involved in cell growth and proliferation
included cyclin D2, FSTL1 and S100A4, confirm-
ing previous observations that cyclin D2 protein
is upregulated in aortic-banded rats (Busk et al.,
2002). FSTL1 and S100A4 are secreted proteins
that have been implicated in cancer cell growth.
Copyright  2004 John Wiley & Sons, Ltd. Comp Funct Genom 2004; 5: 459-470.
464 C. C. Strom et al.
Table
2.
Echocardiography
Weeks
n
AWTd
AWTs
PWTd
PWTs
LVAD
LVAS
FAC
Aortic
banding
6
6
0.237

0.017

0.434

0.025

0.247

0.014

0.426

0.022

0.495

0.034
0.092

0.015
0.820

0.016
Sham
6
6
0.160

0.004
0.318

0.006
0.150

0.003
0.313

0.010
0.513

0.021
0.105

0.011
0.798

0.016
Aortic
banding
12
6
0.252

0.008

0.430

0.016

0.250

0.007

0.432

0.011

0.513

0.026
0.115

0.022
0.779

0.037
Sham
12
6
0.155

0.008
0.325

0.016
0.152

0.009
0.320

0.011
0.480

0.033
0.090

0.024
0.822

0.033
Aortic
banding
16
8
0.270

0.009

0.448

0.019

0.264

0.009

0.444

0.017

0.499

0.037
0.125

0.026
0.757

0.041
Sham
16
6
0.168

0.007
0.330

0.011
0.172

0.007
0.330

0.011
0.428

0.027
0.092

0.015
0.789

0.028
Aortic
banding
30
8
0.290

0.017

0.449

0.018

0.285

0.015

0.449

0.021

0.555

0.043
0.188

0.056
0.697

0.068
Sham
30
6
0.195

0.006
0.332

0.008
0.178

0.007
0.320

0.010
0.513

0.014
0.113

0.015
0.778

0.031
MI
3
17
0.097

0.007

0.101

0.006

0.176

0.008
0.251

0.006

0.826

0.027

0.596

0.025

0.280

0.015

Sham
3
6
0.172

0.005
0.303

0.012
0.157

0.006
0.303

0.015
0.438

0.029
0.108

0.014
0.756

0.021
MI
9
14
0.089

0.007

0.096

0.013

0.167

0.007
0.241

0.015

0.975

0.04

0.756

0.039

0.222

0.030

Sham
9
6
0.163

0.007
0.343

0.007
0.160

0.003
0.347

0.007
0.503

0.018
0.095

0.007
0.812

0.009
Shunt
3
10
0.168

0.007
0.306

0.015
0.172

0.006

0.314

0.012

0.618

0.038

0.206

0.024

0.673

0.023

Sham
3
6
0.152

0.004
0.293

0.011
0.148

0.008
0.275

0.008
0.418

0.015
0.105

0.011
0.751

0.019
Shunt
8
12
0.160

0.006
0.300

0.013
0.159

0.005
0.295

0.011
0.825

0.033

0.295

0.019

0.639

0.025
Sham
8
6
0.165

0.002
0.308

0.014
0.163

0.006
0.303

0.012
0.545

0.026
0.148

0.022
0.729

0.040
Values
are
listed
as
mean

SEM.
Left
ventricular
anterior
wall
thickness
(AWT),
left
ventricular
posterior
wall
thickness
(PWT),
and
left
ventricular
area
(LVA)
were
measured
by
echocardiography
in
a
short
axis
2D
view.
Measurements
were
made
in
both
systole
(-s)
and
diastole
(-d).
Fractional
area
of
change
(FAC)
was
calculated
as
(LVA-d
-
LVA-s/LVA-d)
and
fractional
shortening
as
(LVD-d
-
LVD-s/LVD-d).

p
<
0.05
vs.
sham.
Copyright  2004 John Wiley & Sons, Ltd. Comp Funct Genom 2004; 5: 459-470.
Gene expression in cardiac hypertrophy 465
Table 3. The 139 known genes with common regulation in all hypertrophy models. Some genes are present in more than
one category, but not all categories have been listed of each gene. See Supplemental Table A for a full list of genes by
general functional category and detailed annotations
Category Genes
Signal transduction Annexin-V, scavenger receptor-B2, KCNE1, peptidylglycine -amidating monooxygenase, S100A10, protein
phosphatase-1a, protein kinase inhibitor-, annexin-II, CNP, Sult1a1, Gata4, TSC22, PDE4a, YWK-II, FKBP1a,
dynein, SMARCB1, NPPB, CD151, phosphotidylinositol transfer protein-, MaoA, annexin-I, protein
phosphatase-1b, prostaglandin receptorF2-, Lsamp, NPPA, APP, granulin, CD9, KCNH2, MCP1, Slc3a2, PTPRO,
osteoprotegerin, RNF28, integrin-1, UNC-119, myoinositol monophosphatase-2
Cell growth/proliferation Ndrg4, annexin-V, Btg1, GADD45A, Hsp27, TIMP1, FSTL1, cyclin-D2, ribosomal protein-L6, EGLN3, CSRP2,
S100A4, ribosomal protein-S27, ribosomal protein-L3, granulin, cyclin-D1, Slc3a2, AIF1
Cell death/apoptosis Death-associated kinase-3, GADD45A, Mfge8, EGLN3, biglycan, fibronectin, S100A4, APP, PRG1, Hsp40
homologue-A4, osteoprotegerin, DNAJB5
Development Ndrg4, vesicle-associated membrane protein-5, annexin-II, Gata4, osteopontin, transgelin, EGLN3, Lsamp, CSRP2,
S100A4, PRG1, MCP1, Integrin-1, tropomyosin-4
Defense/inflammation Gpx3, CD1D, Psme1, osteopontin, Hsp27, complement C1-inhibitor, Thy1, Lysozyme, Hsp90A, annexin-I, FCGRT,
KLRG1, MCP1, AIF1, Pai1, complement C1qb, cathepsin-S, Fcgr3
Cytoskeletal/ecm organisation Protease nexin-1, ARGBP2, Actin- skeletal muscle, tubulin-1, CNP, osteopontin, procollagen type 3-1,
transgelin, Hsp27, -actin, TIMP1, cathepsin-L, profilin-1, TIMP2, MYL6, MYL9, biglycan, RLC, fibronectin, -actin
vascular smooth muscle, vimentin, thrombospondin-4, lysyl oxidase, osteoprotegerin, sarcosin, tropomyosin-4, Pai1,
cathepsin-S
Metabolism AQP7, aldehyde dehydrogenase-1a1, Pcyt2, 4-hydroxyphenylpyruvate dioxygenase, ACAT1, 2,4-dienoyl-CoA
reductase, dodecenoyl-CoA--isomerase, Slc25a5, OSCP, trifunctional protein-, trifunctional protein-, PS-PLa1,
GOT1, Sult1a1, GOT2, retinol-binding protein-1, CK-B, ornitine decarboxylase antizyme inhibitor, FACL2, Slc16a1,
cathepsin-L, ferritin light chain, GNPAT, glycogen phosphorylase, SDHA, guanylate kinase 1, NNT, natriuretic
peptide receptor-3, MaoA, enoyl-CoA hydratase-like, phosphofructokinase-muscle, ERp29, BCAT2, succinyl-CoA
ligase, ACAA2, FUCA1, Carbonic anhydrase-2, CK-mit, COX8h, DLAT, ETFA, PDHA2, Slc7a1, AMA-CR,
cathepsin-S, GAMT
Unknown Nuclear receptor binding factor-1, ICT1, major vault protein
Differences in gene expression patterns
between hypertrophic phenotypes
We next looked for the transcriptional responses
specific to each hypertrophy model by asking
which genes showed expression differences bet-
ween the different hypertrophic models, using
ANOVA. This analysis identified 44 genes with
differential expression between the models (Sup-
plemental Table B). The genes represented 40
known genes, two ESTs and two replicates of ANP.
Of these genes, 15 differed only in fold change
between the models but were consistently either up-
or downregulated in all models compared to con-
trols (Supplemental Table B). A hierarchic cluster
analysis of the 44 genes showed tight clustering of
the disease models but separate clustering of the
pharmacologically treated animals (Supplemental
Figure A). Thus, the differences between the mod-
els primarily resulted from differences between the
disease models and the pharmacologically treated
animals. After exclusion of the pharmacologically
treated animals, only seven genes were differen-
tially expressed: Mapk7, skeletal -actin, Csrp2,
acetyl-CoA acyltransferase, ANP (two genes) and
an EST.
Confirmation by real-time PCR
To independently confirm the microarray data,
mRNA levels of eight genes were analysed by real-
time PCR (Figure 1). The real-time PCR analyses
all confirmed the microarray findings, although the
fold change tended to be greater when determined
by real-time PCR.
Discussion
Our work addresses global aspects of gene regu-
lation in surgical and pharmacological models of
cardiac hypertrophy in mammals. Is there a com-
mon set of genes that largely defines cardiac hyper-
trophy in these models? To address this question,
we compared transcriptome responses among five
models of cardiac hypertrophy. We identified a set
of 139 genes and 13 ESTs that were commonly reg-
ulated in cardiac hypertrophy in response to diverse
Copyright  2004 John Wiley & Sons, Ltd. Comp Funct Genom 2004; 5: 459-470.
466 C. C. Strom et al.
Vehicle
Vehicle
Shunt
3-sham
Shunt
3-sham
Shunt
8-sham
Shunt
8-sham
MI
3-sham
MI
3-sham
MI9-sham
MI9-sham
AB-sham
AB-sham
AB12-sham
AB12-sham
AB16-sham
AB16-sham
AB30-sham
AB30-sham
Neonatal
Neonatal
AngII
AngII
Ditpa
Ditpa
Shunt
3
Shunt
3
Shunt
8
Shunt
8
MI
3
MI
3
MI
3
MI
3
MI
9
MI
9
MI
9
MI
9
AB
6
AB
6
AB
12
AB
12
AB
16
AB
16
AB
30
AB
30
AB
30
AB
30
EST
EST
Follistatin-related protein (FSTL1)
EST
Fibronectin
Vimentin
EST
Ribosomal protein L3
Retinol binding protein 1
Ribosomal protein L3
Annexin II
MYL6
Btg1
Integrin-beta1
CD9
Pyruvate dehydrogenase E1 alpha (PDHA2)
Ribosomal protein S27
Adenine nucleotide translocator 2 (Slc25a5)
Granulin
Cathepsin L
Protease nexin-1
S100A10
EST
Annexin 1
Lysyl oxidase
Procollagen type 3 alpha 1
Procollagen type 3 alpha 1
Tubulin-alpha1
Fibronectin
MYL9
MYL9
Slc7a1
Tropomyosin 4 (Tpm4)
Guanylate kinase 1
Annexin V
Annexin V
FKBP1a
Granulin
Ferritin light chain
Profilin1
Vesicle-associated membrane protein (vamp5)
Gata4
Actin-alpha vascular smooth muscle
Ribosomal protein L6
Tubulin-alpha1
ERp29
FCGRT
YWK-II
Death-associated kinase 3
Beta actin
Protease nexin-2 (APP)
Protease nexin-2 (APP)
Protein phosphatase 1a
Protein phosphatase 1b
Heat shock protein 27 (Hsp27)
EST
EST
Prostaglandin receptor F2-alpha
NPPA (ANP)
NPPA (ANP)
NPPA (ANP)
NPPA (ANP)
Biglycan
Actin-alpha skeletal muscle (ACTA1)
NPPB (BNP)
Arg/abl-interacting protein ArgBP2
Cysteine- and glycine-rich protein 2
Biglycan
Cyclin D2
Cyclin D2
PDE4a
Monocarboxylate transporter 1 (Slc16a1)
Lactadherin (Mfge8)
Dynein
Transgelin
Cyclin d1
Phosphotidylinositol transfer protein-beta
PRG1
Thy1
S100A4
Tissue inhibitor of metalloproteinase-1
Tissue inhibitor of metalloproteinase-1
Thrombospondin 4 (Tsp4)
Cyclic nucleotide phosphodiesterase
Osteopontin
TSC-22
Fucosidase-alpha-L-1 (FUCA1)
EST
Tissue inhibitor of metalloproteinase-2
Heat shock protein 90-alpha (H sp90A)
Monocarboxylate transporter 1 (Slc16a1)
Allograft inflammatory factor 1 (AIF1)
Glutathione peroxidase-3 (GPX3)
Glutathione peroxidase-3 (GPX3)
UNC-119
Arg/abl-interacting protein ArgBP2
Potassium channel (KCNE1)
Natriuretic peptide receptor-3 (NPR3)
Protein tyrosine phosphatase, receptor type, O
Osteoprotegerin
Scavenger receptor B2
PRG1
Myosin regulatory light chain
Ornitine decarboxylase antizyme inhibitor
Dihydrolipoamide acetyltransferase (DLAT)
Creatine kinase-B
Ndrg4
EGLN3
phosphate cytidylyltransferase 2 (pcyt2)
EGLN3
Sarcosin
Slc3a2
Peptidylglycine a-amidating monooxygenase
Peptidylglycine a-amidating monooxygenase
Heat shock protein 40 homolog A4
GADD45A
GADD45A
Heat shock protein 27 (Hsp27)
Protein kinase inhibitor alpha
CD151
KLRG1
Complement C1-inhibitor
Aldehyde dehydrogenase-1a1
Monoamine oxidase A (MaoA)
Monoamine oxidase A (MaoA)
EST
Fcgr3
Cathepsin S (CTSS)
Lysozyme
Complement C1qb
PS-PLa1
Plasminogen activator inhibitor-1 (Pai1)
Monocyte chemoattractant protein
Monocyte chemoattractant protein
DNAJB5
SMARCB1
Major vault protein
EST
EST
Aquaporin 7 (Aqp7)
Acetyl-CoA acyltransferase (Acaa2)
Enoyl-CoA hydratase-like
2,4-dienoyl-CoA reductase
Trifunctional protein-beta
Dodecenoyl-CoA-delta-isomerase
Succinyl-CoA ligase (SUCLG1)
Succinate dehydrogenase complex-subunit a
NAD transhydrogenase (NNT)
Fatty acid CoA ligase, long chain 2
Aspartate aminotransferase 1 (GOT1)
CD1D
Acetyl-CoA acyltransferase (Acat1)
Glycogen phosphorylase
Phosphofructokinase-muscle (PFKM)
Dodecenoyl-CoA-delta-isomerase
BCAT2
GNPAT
4-hydroxyphenylpyruvate dioxygenase (hpd)
4-hydroxyphenylpyruvate dioxygenase (hpd)
Myoinositol monophosphatase2
Trifunctional protein-alpha
Proteasome activator subunit alpha (psme1)
Aspartate aminotransferase 2 (GOT2)
EST
EST
ICT1
Ring finger protein 28 (RNF28)
Nuclear receptor binding factor1
COX8h
Guanidinoacetate methyltransferase (GAMT)
Alpha-methylacyl-CoA racemase (AMA-CR)
Lsamp
KCNH2
ETFA
ATP synthase 0 subunit
Creatine kinase-mit.
Creatine kinase-mit.
Sult1a1
Carbonic anhydrase2
-1.35 0.15 1.66
Hypertrophy
N
Control
Vehicle
Vehicle
Shunt
3-sham
Shunt
3-sham
Shunt
8-sham
Shunt
8-sham
MI
3-sham
MI
3-sham
MI9-sham
MI9-sham
AB-sham
AB-sham
AB12-sham
AB12-sham
AB16-sham
AB16-sham
AB30-sham
AB30-sham
Neonatal
Neonatal
AngII
AngII
Ditpa
Ditpa
Shunt
3
Shunt
3
Shunt
8
Shunt
8
MI
3
MI
3
MI
3
MI
3
MI
9
MI
9
MI
9
MI
9
AB
6
AB
6
AB
12
AB
12
AB
16
AB
16
AB
30
AB
30
AB
30
AB
30
EST
EST
Follistatin-related protein (FSTL1)
EST
Fibronectin
Vimentin
EST
Ribosomal protein L3
Retinol binding protein 1
Ribosomal protein L3
Annexin II
MYL6
Btg1
Integrin-beta1
CD9
Pyruvate dehydrogenase E1 alpha (PDHA2)
Ribosomal protein S27
Adenine nucleotide translocator 2 (Slc25a5)
Granulin
Cathepsin L
Protease nexin-1
S100A10
EST
Annexin 1
Lysyl oxidase
Procollagen type 3 alpha 1
Procollagen type 3 alpha 1
Tubulin-alpha1
Fibronectin
MYL9
MYL9
Slc7a1
Tropomyosin 4 (Tpm4)
Guanylate kinase 1
Annexin V
Annexin V
FKBP1a
Granulin
Ferritin light chain
Profilin1
Vesicle-associated membrane protein (vamp5)
Gata4
Actin-alpha vascular smooth muscle
Ribosomal protein L6
Tubulin-alpha1
ERp29
FCGRT
YWK-II
Death-associated kinase 3
Beta actin
Protease nexin-2 (APP)
Protease nexin-2 (APP)
Protein phosphatase 1a
Protein phosphatase 1b
Heat shock protein 27 (Hsp27)
EST
EST
Prostaglandin receptor F2-alpha
NPPA (ANP)
NPPA (ANP)
NPPA (ANP)
NPPA (ANP)
Biglycan
Actin-alpha skeletal muscle (ACTA1)
NPPB (BNP)
Arg/abl-interacting protein ArgBP2
Cysteine- and glycine-rich protein 2
Biglycan
Cyclin D2
Cyclin D2
PDE4a
Monocarboxylate transporter 1 (Slc16a1)
Lactadherin (Mfge8)
Dynein
Transgelin
Cyclin d1
Phosphotidylinositol transfer protein-beta
PRG1
Thy1
S100A4
Tissue inhibitor of metalloproteinase-1
Tissue inhibitor of metalloproteinase-1
Thrombospondin 4 (Tsp4)
Cyclic nucleotide phosphodiesterase
Osteopontin
TSC-22
Fucosidase-alpha-L-1 (FUCA1)
EST
Tissue inhibitor of metalloproteinase-2
Heat shock protein 90-alpha (H sp90A)
Monocarboxylate transporter 1 (Slc16a1)
Allograft inflammatory factor 1 (AIF1)
Glutathione peroxidase-3 (GPX3)
Glutathione peroxidase-3 (GPX3)
UNC-119
Arg/abl-interacting protein ArgBP2
Potassium channel (KCNE1)
Natriuretic peptide receptor-3 (NPR3)
Protein tyrosine phosphatase, receptor type, O
Osteoprotegerin
Scavenger receptor B2
PRG1
Myosin regulatory light chain
Ornitine decarboxylase antizyme inhibitor
Dihydrolipoamide acetyltransferase (DLAT)
Creatine kinase-B
Ndrg4
EGLN3
phosphate cytidylyltransferase 2 (pcyt2)
EGLN3
Sarcosin
Slc3a2
Peptidylglycine a-amidating monooxygenase
Peptidylglycine a-amidating monooxygenase
Heat shock protein 40 homolog A4
GADD45A
GADD45A
Heat shock protein 27 (Hsp27)
Protein kinase inhibitor alpha
CD151
KLRG1
Complement C1-inhibitor
Aldehyde dehydrogenase-1a1
Monoamine oxidase A (MaoA)
Monoamine oxidase A (MaoA)
EST
Fcgr3
Cathepsin S (CTSS)
Lysozyme
Complement C1qb
PS-PLa1
Plasminogen activator inhibitor-1 (Pai1)
Monocyte chemoattractant protein
Monocyte chemoattractant protein
DNAJB5
SMARCB1
Major vault protein
EST
EST
Aquaporin 7 (Aqp7)
Acetyl-CoA acyltransferase (Acaa2)
Enoyl-CoA hydratase-like
2,4-dienoyl-CoA reductase
Trifunctional protein-beta
Dodecenoyl-CoA-delta-isomerase
Succinyl-CoA ligase (SUCLG1)
Succinate dehydrogenase complex-subunit a
NAD transhydrogenase (NNT)
Fatty acid CoA ligase, long chain 2
Aspartate aminotransferase 1 (GOT1)
CD1D
Acetyl-CoA acyltransferase (Acat1)
Glycogen phosphorylase
Phosphofructokinase-muscle (PFKM)
Dodecenoyl-CoA-delta-isomerase
BCAT2
GNPAT
4-hydroxyphenylpyruvate dioxygenase (hpd)
4-hydroxyphenylpyruvate dioxygenase (hpd)
Myoinositol monophosphatase2
Trifunctional protein-alpha
Proteasome activator subunit alpha (psme1)
Aspartate aminotransferase 2 (GOT2)
EST
EST
ICT1
Ring finger protein 28 (RNF28)
Nuclear receptor binding factor1
COX8h
Guanidinoacetate methyltransferase (GAMT)
Alpha-methylacyl-CoA racemase (AMA-CR)
Lsamp
KCNH2
ETFA
ATP synthase 0 subunit
Creatine kinase-mit.
Creatine kinase-mit.
Sult1a1
Carbonic anhydrase2
-1.35 0.15 1.66
Hypertrophy
N
Control
Figure
2.
Hierarchic
cluster
analysis
based
on
179
genes
with
a
unified
gene
expression
response
in
different
phenotypes
of
cardiac
hypertrophy.
The
distances
between
genes
were
calculated
as
the
angle
between
the
vectors
constituted
by
the
log
2
fold
changes
and
a
weighted
pair-group
average
linkage
was
used
for
clustering.
Each
column
represents
a
tissue
sample
and
each
row
represents
a
gene.
Increasing
intensity
of
red
indicates
upregulation
and
green
downregulation.
Genes
with
no
change
in
expression
are
displayed
in
a
light
shade
of
green
as
illustrated
in
the
colour
bar
at
the
bottom
of
the
figure.
Genes
with
the
most
similar
expression
profiles
across
experiments
are
clustered
together.
The
dendrogram
shows
gene
clustering
based
on
all
the
samples.
The
clustering
algorithm
was
applied
to
the
genes
only.
Gene
names
are
listed
to
the
right
and
sample
identity
at
the
top.
Neonatal
samples
were
included
for
comparison.
Abbreviations:
mi,
myocardial
infarction;
ab,
aortic
banding;
ang2,
angiotensin
II;
Ditpa,
3,5-diiodothyropropionic
acid.
The
numbers
after
the
sample
names
correspond
to
the
number
of
weeks
after
surgery
that
the
samples
were
taken
Copyright  2004 John Wiley & Sons, Ltd. Comp Funct Genom 2004; 5: 459-470.
Gene expression in cardiac hypertrophy 467
Down-regulated
14%
6%
77%
3%
Up-regulated
8%
10%
13%
13%
14%
23%
19%
metabolism
signal transduction
cytoskeletal/ecm organisation
defence/inflammation
cell growth/proliferation
development
cell death/apoptosis
26%
22%
15%
12%
10%
8%
6%
All
Figure 3. Known genes (n = 139) with common expression in all hypertrophic models were categorized based on function,
as determined from database searches. Genes involved in the cytoskeleton/extracellular matrix (ecm) organization, cell
death/apoptosis, and cell growth/proliferation were exclusively upregulated. The vast majority of downregulated genes
were involved in the -oxidation of fatty acids
and naturally occurring pathological stimuli, such
as pressure overload, volume overload and myocar-
dial infarction. Overall, this outcome is important
for two reasons. First, these transcriptome changes
are likely to signify molecular events necessary
for, or a consequence of, the hypertrophic process
in these models. Second, using a global approach,
our data support and expand the notion of a uni-
versal gene program underlying cardiac hypertro-
phy in response to multiple diversified stimuli.
This paradigm was previously based on transcrip-
tional changes in a small set of genes, including
immediate early genes, ANP, BNP, and sarcomer
genes constituting the socalled `fetal gene program'
(Chien et al., 1991).
Many genes in our core set including ANP, BNP,
-actin, procollagen, fibronectin, GATA4, riboso-
mal proteins, biglycan, vimentin, Hsp27, TSC22,
PAI1, and osteopontin were consistently regu-
lated in previous microarray studies analyzing the
myocardium after MI in rats, in failing hearts from
hypertensive rats, in neonatal and adult mice and
in failing and hypertrophic human hearts (Sup-
plemental table A) (Anversa et al., 1983; Barrans
et al., 2002; Friddle et al., 2000; Hwang et al.,
2000, 2002; Schoenfeld et al., 1998; Sehl et al.,
Copyright  2004 John Wiley & Sons, Ltd. Comp Funct Genom 2004; 5: 459-470.
468 C. C. Strom et al.
2000; Stanton et al., 2000; Yang et al., 2000).
Remarkably, although these studies employed dif-
ferent experimental setups and microarray tech-
nologies, including self-made or customized arrays
containing cDNAs or oligonucleotides, and dif-
ferent clustering algorithms, they reported many
similar transcriptome changes, of which the most
highlighted were upregulation of ecm proteins
(Anversa et al., 1983; Barrans et al., 2002; Friddle
et al., 2000; Hwang et al., 2000, 2002; Schoen-
feld et al., 1998; Sehl et al., 2000; Stanton et al.,
2000; Yang et al., 2000). Employing two hormone-
induced hypertrophy models in mice, Friddle et al.
(2000) found 38 annotated gene changes, of which
16 was included in our common hypertrophy
genes. Using Affymetrix arrays, Weinberg et al.
(2003) reported increased expression of a number
of genes in response to acute and chronic pressure
overload in male and female mice. Of 12 483 tested
genes, 68 genes were upregulated in males and
females, of which several were consistent with our
results, including ornitine decarboxylase antizyme
inhibitor, fibronectin, tissue-inhibitor of metallo-
proteinase, procollagen, glutatione peroxidase 3,
nexin, thrombospondin, biglycan, skeletal -actin,
-actin and ANP. However, to the best of our
knowledge no other study compares transcriptome
changes across the multiple forms of hypertrophy
we here employed. Furthermore, the large sample
set (n = 46) allowed us to use the Bonferroni cor-
rection that reduces the amount of false positives.
We employed diverse pathological stimuli to
induce hypertrophy in the animals, but only a few
genes were differentially expressed between the
models (44 genes). The transcriptome differences
between models were primarily between the sur-
gical models and the hormone-stimulated models,
which could be due to the selective nature of hor-
mone stimulation as compared to more complex
stimuli in the surgical models. Perhaps predictably,
hormone infusion might be less suited for deter-
mination of genes regulated by pathophysiological
processes in vivo than actual disease models.
Although these findings indicate that overall the
gene expression in the different surgical models is
more similar than different, it should be taken into
consideration that this study was designed primar-
ily to identify a common trunk of genes across
models. The statistical power declines when sub-
grouping samples, resulting in an increased risk
of type 2 errors, i.e. missing genes that actually
change. In addition, we used a conservative statisti-
cal correction (Bonferroni) to keep type 1 statistical
error (false positives) low despite the multiple com-
parisons (total < 0.05). Had we used a less strin-
gent statistical approach, we could have found a
higher number of transcriptome changes between
the models.
Our data suggest that the essential attributes
of hypertrophic remodelling of the myocardium
include:
1. Activation of growth-inducing molecules, inclu-
ding enhanced expression of growth factors
(Mcp, S100A4, Mgfe8 and granulin, both EGF-
like proteins), cyclin D2, FSTL1 (growth factor
implicated in cancer), TGF signalling proteins,
and several ribosomal proteins.
2. Activation of multiple signalling modules, inclu-
ding those leading to: (a) accumulation of inos-
itol phosphates (enhanced expression of recep-
tors that stimulate PI accumulation), PI trans-
fer protein-B (engaged in phospholipid trans-
port), CD9 and CD151 (link PKC to specific
integrins), and myoinositol monophosphatase,
and (b) changes in Ca2+
handling, ion-channels
(KCNE1 and KCNH2) and channel-associated
proteins, dubbed annexins.
3. Enhanced remodelling through ecm and cytos-
keletal systems, including cytoskeletal-associa-
ted proteins, such as myosin and dynein (cytos-
keletal molecular motors), vimentin, SMAR-
CH1 (an actin-dependent regulator of chro-
matin remodelling), fibronectin, ecm proteases,
transgelin (participates in cytoskeleton assem-
bly), ArgBP2 (a Src-3 domain containing pro-
tein that binds to sarcomer elements) and inte-
grin are enhanced.
4. Engagement of defence and inflammation pro-
teins related to coagulation and lymphocyte acti-
vation were enhanced, such as annexins, pro-
tease nexin-1, pai-1, complement C1 inhibitor,
and complement C1gb).
5. Changes in genes involved in energy metabolism
suggest a shift away from lipids as the main
energy source in the myocardium, consistent
with previous reports (Stanton et al., 2000;
Yang et al., 2000). Several key regulatory genes
involved in fatty acid oxidation pathways,
e.g. acetyl-CoA acyltransferase, dienoyl CoA-
reducase, fatty acid CoA-ligase, dodecenoyl
CoA-isomerase, and enoyl CoA-hydratase were
Copyright  2004 John Wiley & Sons, Ltd. Comp Funct Genom 2004; 5: 459-470.
Gene expression in cardiac hypertrophy 469
repressed, as depicted in Supplemental Figure B.
Many genes involved in the mitochondrial respi-
ratory chain, e.g. OSCP, SDHA, NNT, COX8h
and ETFA, were downregulated, indicating a
general defect in energy metabolism in hyper-
trophic hearts, as also noted by others (Stanton
et al., 2000; Yang et al., 2000).
A notable finding was that upregulation of three
annexins (I, II and V), members of a family of pro-
teins that have anticoagulant effects, inhibits leuko-
cyte infiltration, and form Ca2+
channels. Annexin
upregulation might have beneficial effects. Annexin
I peptides protect against ischaemia-reperfusion
injury of the myocardium, most likely by inhibit-
ing leukocyte infiltration (La et al., 2001). Annexin
channel formation might be required for changes in
Ca2+
homeostasis and gene expression in hyper-
trophic cardiomyocytes. In hypertrophic chron-
docytes, annexin-mediated Ca2+
influx activates
gene expression of Cbfa1, ATPase and osteocal-
cin, which are differentiation markers in these cells
(Wang et al., 2003).
Another interesting finding was increased expres-
sion of granulin, a growth factor that resembles the
EGF/TGF family, although granulin does not acti-
vate EGF receptors (Bateman et al., 1998). Gran-
ulins regulate differentiation/proliferation of mul-
tiple cells, including epithelial and haematopoi-
etic cells and carcinoma and breast cancer cell
lines. Granulin was found upregulated in human
glioblastomas in a cDNA microarray study (Liau
et al., 2000). Further work will reveal whether
granulin plays a role in cardiomyocyte hyper-
trophy. It should be noted that TSC-22 (TGF-
controlled transcription factor), FSTL1, and PAI-1
(TGF-induced effectors) were upregulated, sug-
gesting that activation of TGF family signalling
is involved in hypertrophy, as also noted by others
(Stanton et al., 2000).
In conclusion, we have identified a set of genes
common to several different clinically relevant
phenotypes of cardiac hypertrophy. These genes
are likely to be central to hypertrophic phenotype,
irrespective of the initiating cause.
Acknowledgements
We thank Paul C. Simpson, UCSF, for critical reading of the
manuscript, and Bettina Collin, Pernille Gundelach, Tordis
Christiansen and Katrine Kastberg for technical assistance.
The work was supported by the John and Birthe Meyer
Foundation; The Danish Heart Foundation (Grant Nos
01-1-2-59-22907, 99-1-2-31-22684); The Villadsen Family
Foundation; The Foundation of 17.12.1981, University
of Copenhagen, Rigshospitalet; and the Danish National
Research Foundation.
References
Agabiti-Rosei E, Muiesan ML. 1997. Prognostic significance of
left ventricular hypertrophy regression. Adv Exp Med Biol 432:
199-205.
Anversa P, Levicky V, Beghi C, McDonald SL, Kikkawa Y.
1983. Morphometry of exercise-induced right ventricular
hypertrophy in the rat. Circ Res 52: 57-64.
Aronow BJ, Toyokawa T, Canning A, et al. 2001. Divergent
transcriptional responses to independent genetic causes of
cardiac hypertrophy. Physiol Genom 6: 19-28.
Barrans JD, Allen PD, Stamatiou D, Dzau VJ, Liew CC. 2002.
Global gene expression profiling of end-stage dilated
cardiomyopathy using a human cardiovascular-based cDNA
microarray. Am J Pathol 160: 2035-2043.
Bateman A, Bennett HJP. 1998. Granulins: the structure and
function of a emerginc family of growth factores. J Endocrinol
158: 145-151.
Busk PK, Bartkova J, Strom CC, et al. 2002. Involvement of
cyclin D activity in left ventricle hypertrophy in vivo and
in vitro. Cardiovasc Res 56: 64-75.
Chien KR, Knowlton KU, Zhu H, Chien S. 1991. Regulation
of cardiac gene expression during myocardial growth and
hypertrophy: molecular studies of an adaptive physiologic
response. FASEB J 5: 3037-3046.
Friddle CJ, Koga T, Rubin EM, Bristow J. 2000. Expression
profiling reveals distinct sets of genes altered during induction
and regression of cardiac hypertrophy. Proc Natl Acad Sci USA
97: 6745-6750.
Golub TR, Slonim DK, Tamayo P, et al. 1999. Molecular
classification of cancer: class discovery and class prediction by
gene expression monitoring. Science 286: 531-537.
Hwang DM, Dempsey AA, Lee CY, Liew CC. 2000. Identifica-
tion of differentially expressed genes in cardiac hypertrophy by
analysis of expressed sequence tags. Genomics 66: 1-14.
Hwang JJ, Allen PD, Tseng GC, et al. 2002. Microarray gene
expression profiles in dilated and hypertrophic cardiomyopathic
end-stage heart failure. Physiol Genom 10: 31-44.
La M, D'Amico M, Bandiera S, et al. 2001. Annexin 1 peptides
protect against experimental myocardial ischemia-reperfusion:
analysis of their mechanism of action. FASEB J 15: 2247-2256.
Li C, Hung Wong W. 2001a. Model-based analysis of oligonu-
cleotide arrays: model validation, design issues and standard
error application. Genome Biol 2.
Li C, Wong WH. 2001b. Model-based analysis of oligonucleotide
arrays: expression index computation and outlier detection. Proc
Natl Acad Sci USA 98: 31-36.
Liau LM, Lallone RL, Seitz RS, et al. 2000. Identification of a
human glioma-associated growth factor gene, granulin, using
differential immunoadsorption. Cancer Res 60: 1353-1360.
Roberts CJ, Nelson B, Marton MJ, et al. 2000. Signalling and
circuitry of multiple MAPK pathways revealed by a matrix of
global gene expression profiles. Science 287: 873-880.
Copyright  2004 John Wiley & Sons, Ltd. Comp Funct Genom 2004; 5: 459-470.
470 C. C. Strom et al.
Schoenfeld JR, Vasser M, Jhurani P, et al. 1998. Distinct
molecular phenotypes in murine cardiac muscle development,
growth, and hypertrophy. J Mol Cell Cardiol 30: 2269-2280.
Sehl PD, Tai JT, Hillan KJ, et al. 2000. Application of cDNA
microarrays in determining molecular phenotype in cardiac
growth, development, and response to injury. Circulation 101:
1990-1999.
Stanton LW, Garrard LJ, Damm D, et al. 2000. Altered patterns
of gene expression in response to myocardial infarction. Circ
Res 86: 939-945.
Wang W, Xu J, Kirsch T. 2003. Annexin-mediated Ca2+
influx
regulates growth plate chondrocyte maturation and apoptosis. J
Biol Chem 278: 3762-3769.
Weinberg EO, Mirotsou M, Gannon J, et al. 2003. Sex dependence
and temporal dependence of the left ventricular genomic
response to pressure overload. Physiol Genom 12: 113-127.
Wollert KC, Taga T, Saito M, et al. 1996. Cardiotrophin-1
activates a distinct form of cardiac muscle cell hypertrophy.
Assembly of sarcomeric units in series VIA gp130/leukemia
inhibitory factor receptor-dependent pathways. J Biol Chem 271:
9535-9545.
Yang J, Moravec CS, Sussman MA, et al. 2000. Decreased
SLIM1 expression and increased gelsolin expression in failing
human hearts measured by high-density oligonucleotide arrays.
Circulation 102: 3046-3052.
Copyright  2004 John Wiley & Sons, Ltd. Comp Funct Genom 2004; 5: 459-470.

				
